# Unexpected Anal Squamous Cells Carcinoma after Open Hemorrhoidectomy

**DOI:** 10.1155/2015/616274

**Published:** 2015-04-02

**Authors:** Navarra Luca, Abruzzese Valentina, Sista Federico, Pietroletti Renato

**Affiliations:** ^1^Department of Surgery, SS. Trinità Hospital, Via Enrico Berlinguer, 65026 Popoli, Italy; ^2^Dipartimento di Scienze Cliniche Applicate e Biotecnologie, University of L'Aquila, Viale dell'Ospedale Edificio Delta 6, 67100 L'Aquila, Italy; ^3^Department of Surgery, University of L'Aquila, Viale dell'Ospedale Edificio Delta 6, 67100 L'Aquila, Italy

## Abstract

We report a case of unexpected anal squamous cells carcinoma found in hemorrhoidectomy specimen. The patient had a 3-year history of prolapsing hemorrhoids. A prolapsing hemorrhoid was present at eleven o'clock in lithotomy. Milligan-Morgan was performed and gross examination of the specimen was unremarkable. Histopathologic evaluation showed noninvasive squamous cells carcinoma. The present case report evidences the opportunity of routine histopathologic analysis of hemorrhoidal specimens particularly in case of long-standing prolapse. Questions arise in the option of those techniques where no specimens are collected or tissue is excised far from deceased area.

## 1. Introduction

Anal cancer is a rare neoplasia; the reported frequency in literature ranges between 1 and 2% [1–3], but its incidence has increased considerably during past decades [4]. Clinical diagnosis can be easily made by physical examination just at an advanced stage, secondary to the aggressive local growth and a higher T stage at presentation. In fact, we can find more concerning symptoms for cancer, such as weight loss, change in stool caliber, constipation, or fecal incontinence [3].

Early symptoms of anal cancer may be difficult to distinguish clinically from hemorrhoids and other benign conditions such as anal fissure, fistula in ano, condylomas, or perianal abscesses. Therefore anal cancer diagnosis is not rarely made after pathologic examination of a specimen taken for treatment of another condition.

Hemorrhoidectomy is a frequently performed operation and the necessity of pathologic evaluation of specimens is still controversial. We present a case of noninvasive squamous cells carcinoma discovered after surgical treatment of haemorrhoids as an example of why it is essential to send all such specimens to the pathologist.

## 2. Case Report

A 48-year-old woman with an unremarkable past medical history presented to our surgical department with a 3-year history of anal bleeding, anal hitch, and prolapse. The patient was initially treated conservatively but in spite of these treatments, symptoms did not regress. Therefore surgery was planned.

Physical examination was unremarkable. Anorectal examination showed a normal perianal skin, but a 4 cm solitary prolapsing hemorrhoidal node at eleven o'clock in lithotomy was present without signs of thrombosis. On Valsalva maneuver, the patient presented minimal descending perineum. Digital anal examination confirmed internal hemorroidal nodule with soft consistency. Any malignant features, such as induration or ulceration, were detected.

**Figure 1:**
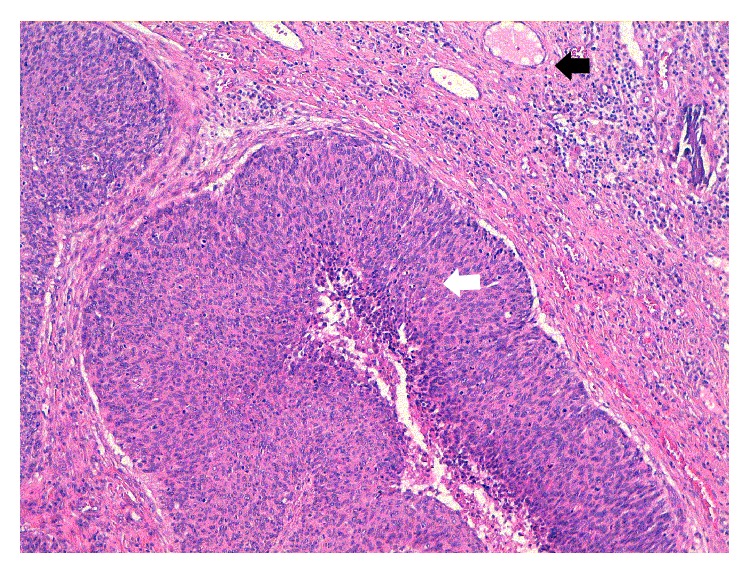
Histopathologic report: noninvasive squamous cells carcinoma (white arrow) without angiolymphatic invasion (black arrow).

Anoscopy confirmed the presence of solitary bulging and prolapsing hemorrhoidal node. Excisional hemorrhoidectomy was performed under local anesthesia. Patient was discharged after a few hours. Gross examination of the specimen was unremarkable. The histopathologic report showed noninvasive squamous cells carcinoma (white arrow) without angiolymphatic invasion (black arrow) (Figure 1).

A metastatic workup revealed normal CT scans of the chest, abdomen, and pelvis. A 4 cm lymph node has been found in the right inguinal region. Surgical and oncological follow-up every 3 months was performed for 27 months. Patient healed without evidence of stricture or local infection or recurrence. The complete absence of hypertrophic node to the next CT scan control and the patient survival at more than 2 years without adjuvant therapies demonstrate the inflammatory origin of this hypertrophic lymph node.

## 3. Discussion

Hemorrhoids are common in western countries, affecting up to 86% of general population [5]. Hemorrhoids are listed among risk factors for anal cancer; long-lasting hemorrhoids, in fact, may act like chronic inflammatory stimulus, which may turn into squamous cell carcinomas [6]. Unexpected anal carcinoma, found after hemorrhoidectomy, is a very rare occurrence; the reported frequency of unrecognized neoplasia found on surgical specimens derived from minor surgery of the anal canal ranges between 0,2% and 4,4% [1, 5, 7].

Given the relative rarity of anal cancer, the only moderate excess long term risk, and the elevated cost of routine histopathologic examination on hemorrhoidectomy specimens, selective rather than routine pathologic evaluation is recommended by some authors. They suggest paying special attention to tender and indurated lesions that should be manually inspected by the operating surgeon: any suspicious areas should be sent for microscopic evaluation [2, 3, 8, 9]. In our case any macroscopic induration or ulceration, as malignant signs, was detected.

In literature we find authors suggesting opportunity of routine pathologic examination of all tissues obtained by hemorrhoidectomy [1, 5, 10, 11].

In this respect the development of new methods for a surgical treatment of hemorrhoids should be taken into account; Stapled Hemorrhoidectomy (Longo procedure) and Transanal Hemorrhoidal Dearterialization (THD) procedure do not allow histopathological examination of hemorrhoidal tissue. In fact in the first surgical method the histopathological examination is made on rectal mucosa and not on the hemorrhoidal node. In the latter, excision of the node is not required.

The present case reported puts the attention on two questions, on one hand the opportunity of routine histopathologic analysis of specimens taken at Milligan-Morgan procedure and, on the other hand, the impossibility to examine hemorrhoidal specimens derived from Longo or THD procedure.

So far the review of the literature does not support routine histopathologic examination of hemorrhoidectomy specimens [2, 3, 8]. However our case demonstrates that full microscopic evaluation of hemorrhoidal specimen can be fundamental to discover unsuspected anal carcinoma.
